# Supplementary material for: Malassezia sympodialis Mala s 1 allergen is a potential KELCH protein that cross reacts with human skin
Source: FEMS Yeast Res. 2023 May 15;23:foad028. doi: 10.1093/femsyr/foad028 (PMC10281499; doi:10.1093/femsyr/foad028)
Supplement: foad028_Supplemental_Files [file foad028_supplemental_files.zip › Mala s 1 Suppl figures combined.pdf]

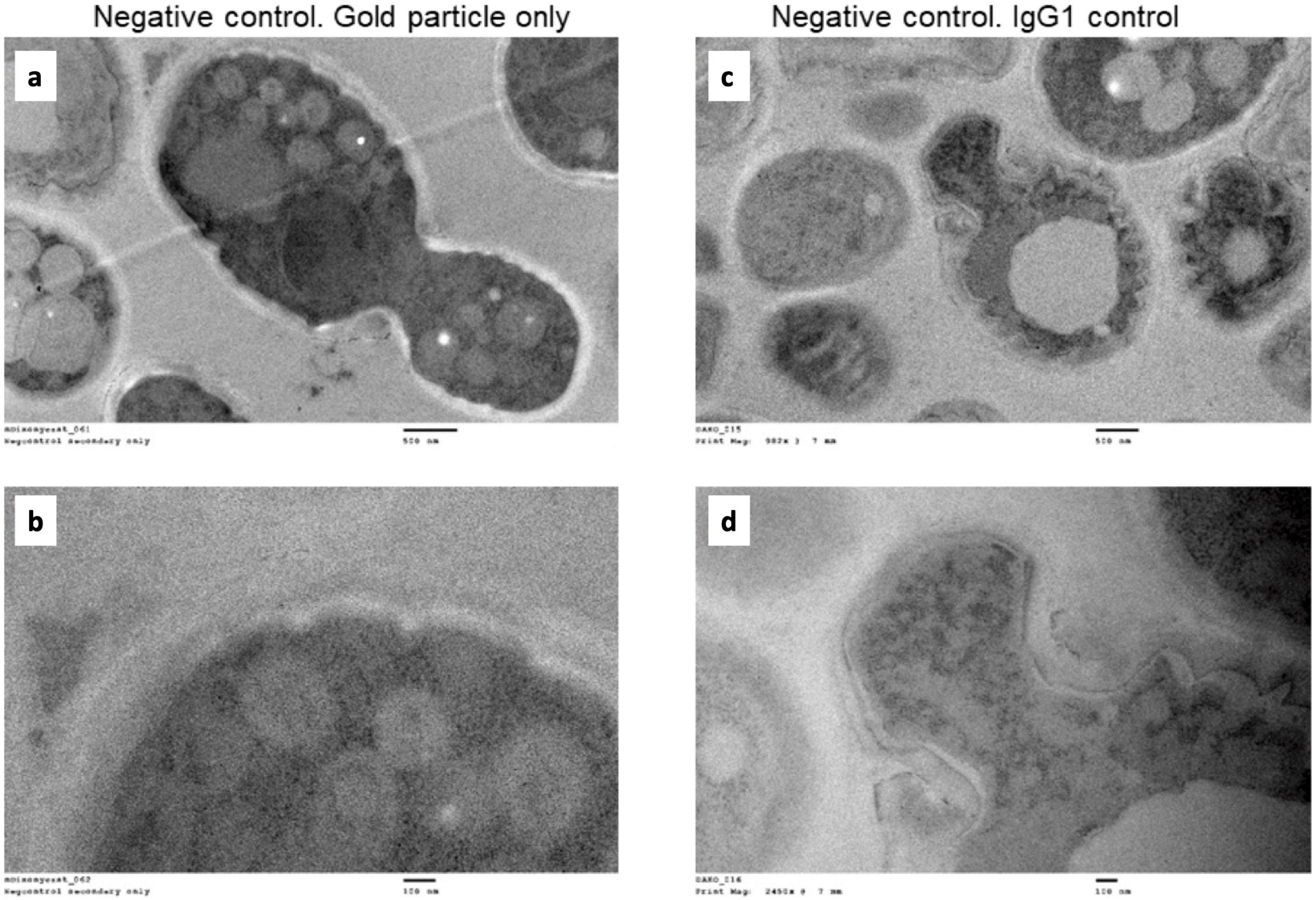
Negative control. Gold particle only
Negative control. IgG1 control
a
c
b
d
500 nm
500 nm
100 nm
100 nm

Figure S1

Minimal effective concentration after 96 h of incubation

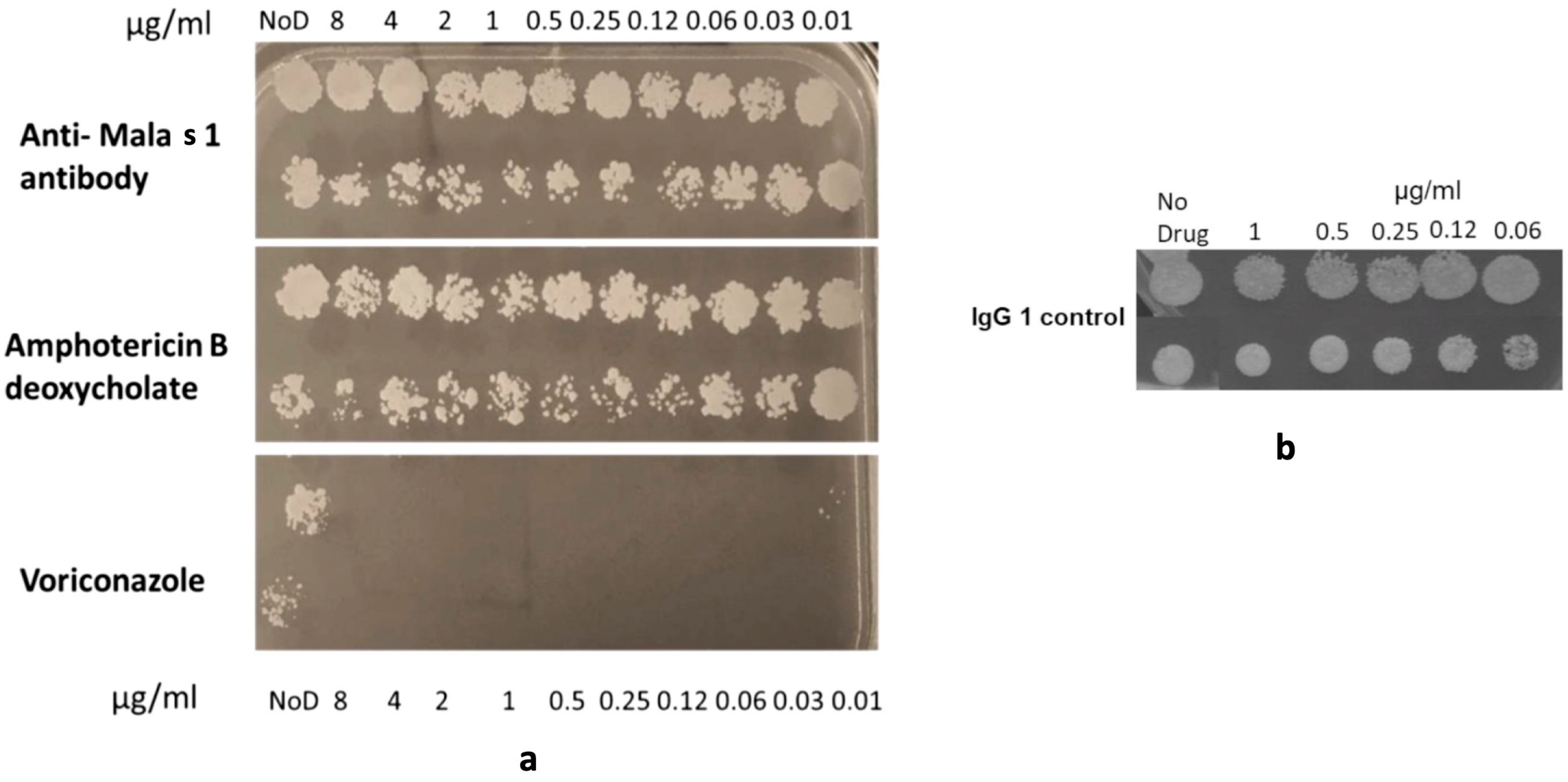

Figure S2

Non-infected human skin (donors 1, 2, 4)

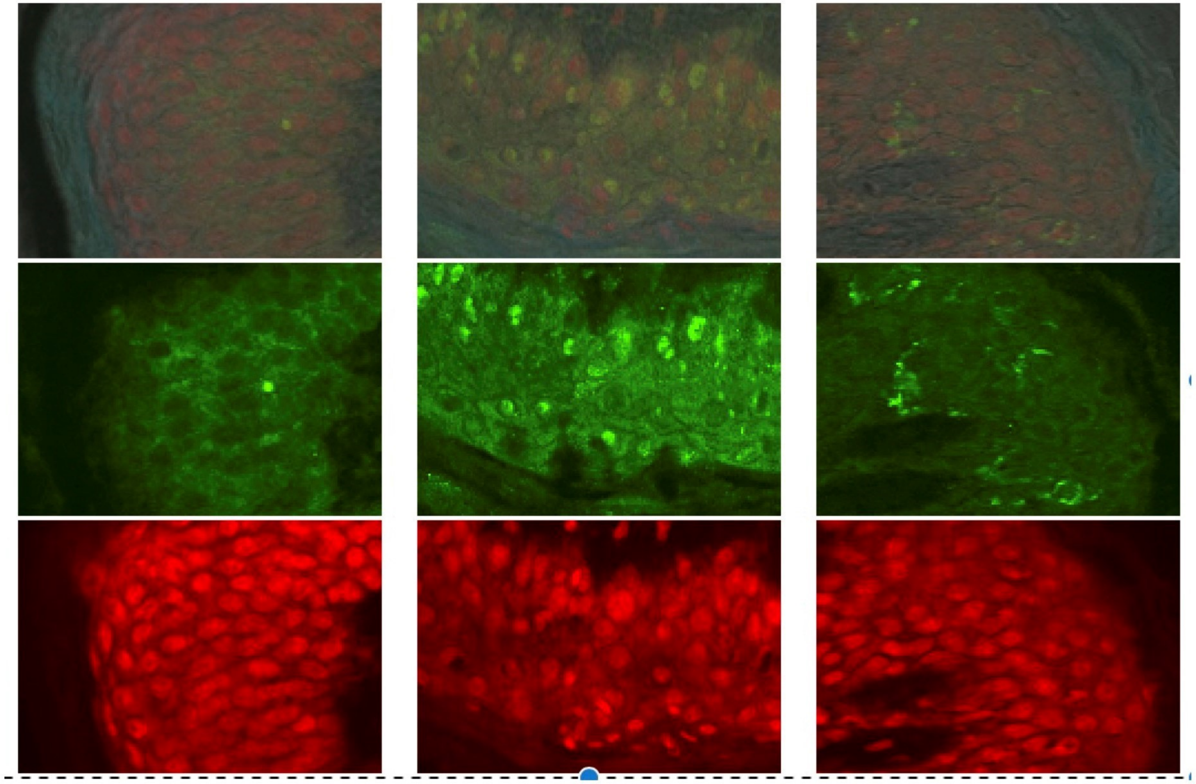

Figure S3

a

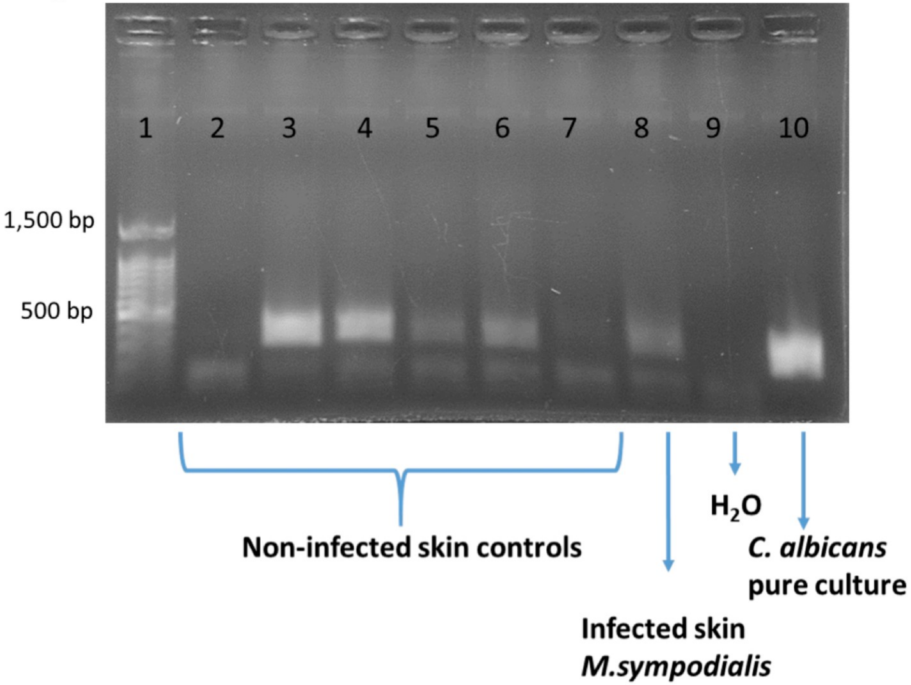

b

Lane	Sample	Amplified	ID in sequencing
1	100 bp ladder		
2	Uninfected skin (3-18)	NO	
3	Uninfected skin (11-17)	YES	2/5 *Sarocladium kiliense*<br>3/5 Uncultured fungi
4	Uninfected skin (4-17)	YES	1/5 *M. globosa*,<br>2/5 *S. schenckii*,<br>2/5 *Sarocladium kiliense*
5	Uninfected (1-18)	YES	3/5 *C. albicans*<br>2/5 no inserts
6	Uninfected skin (6-18)	YES	2 plasmids with unspecific sequence inserts
7	Uninfected skin (5-17)	NO	
8	*M. sympodialis* Infected skin	Yes	2 plasmids with *M. sympodialis* inserts
9	Water	No	
10	*C. albicans* ATCC 90028	Yes	*C. albicans*, 3/3 plasmids

Figure S4
