# Supplementary figures and images for: Malassezia sympodialis Mala s 1 allergen is a potential KELCH protein that cross reacts with human skin
Source: FEMS Yeast Res. 2023 May 15;23:foad028. doi: 10.1093/femsyr/foad028 (PMC10281499; doi:10.1093/femsyr/foad028)

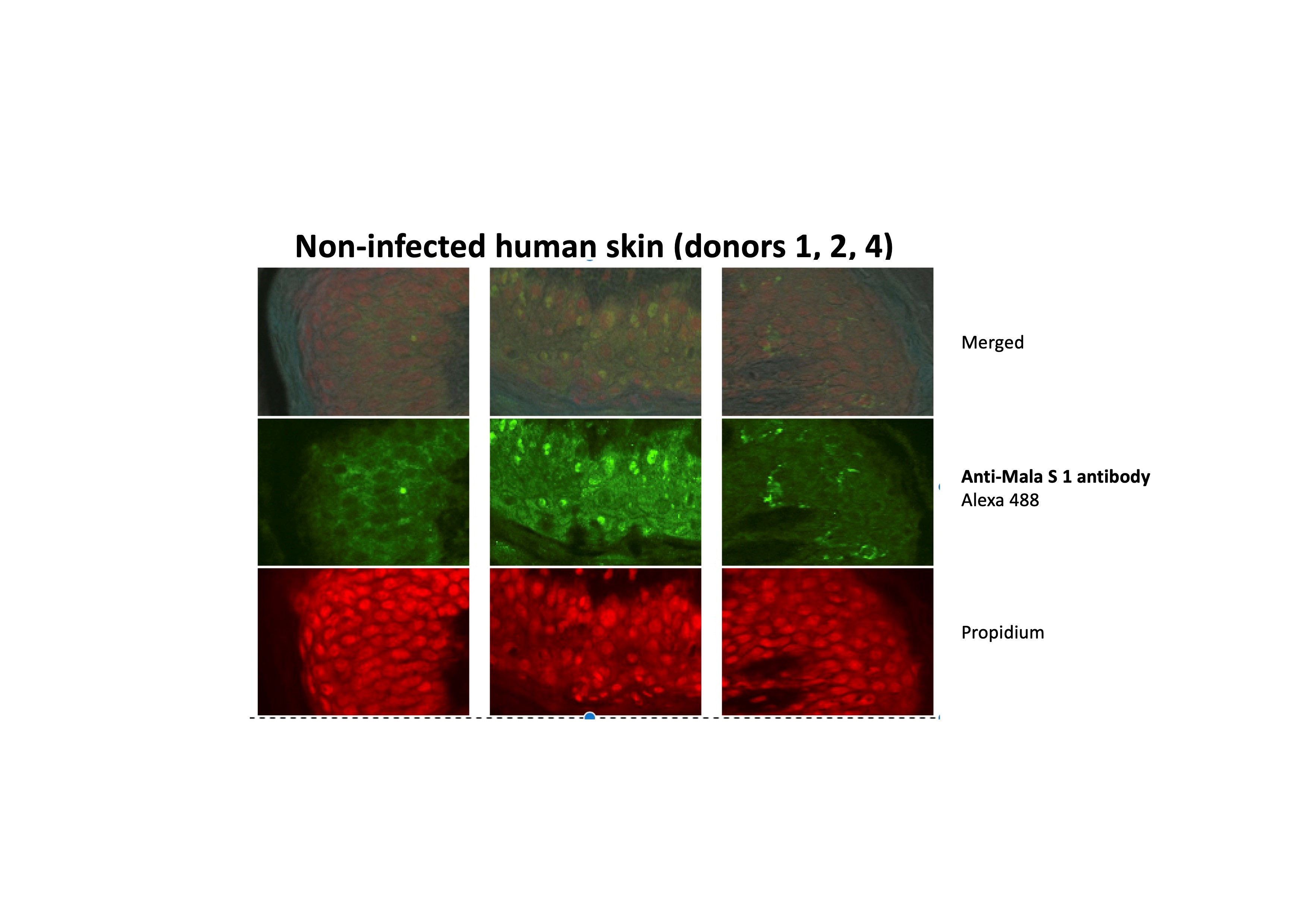

Supplement: foad028_Supplemental_Files [file foad028_supplemental_files.zip › Fig 3 Suppl corrected.jpg]

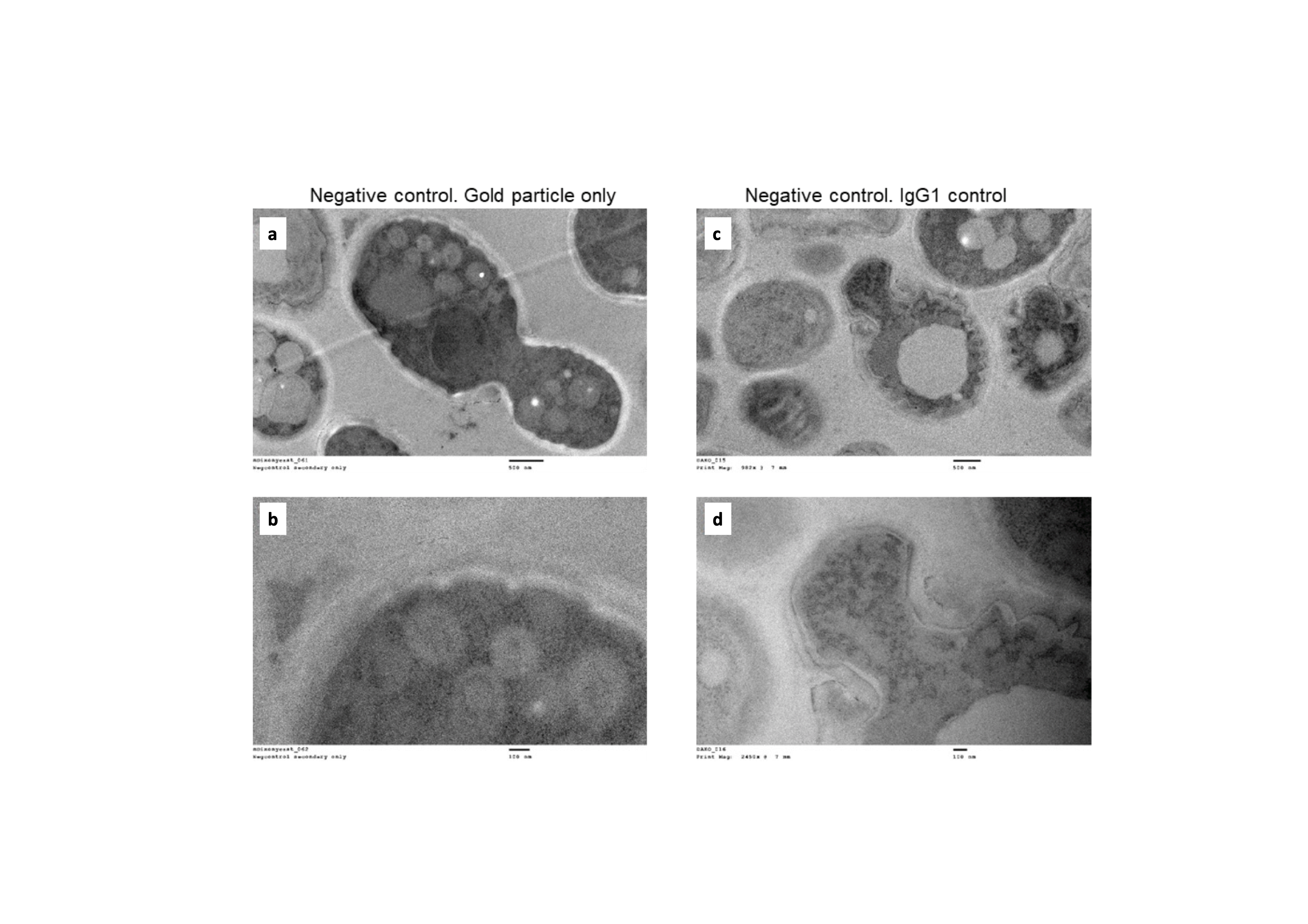

Supplement: foad028_Supplemental_Files [file foad028_supplemental_files.zip › Sup Fig 1.png]

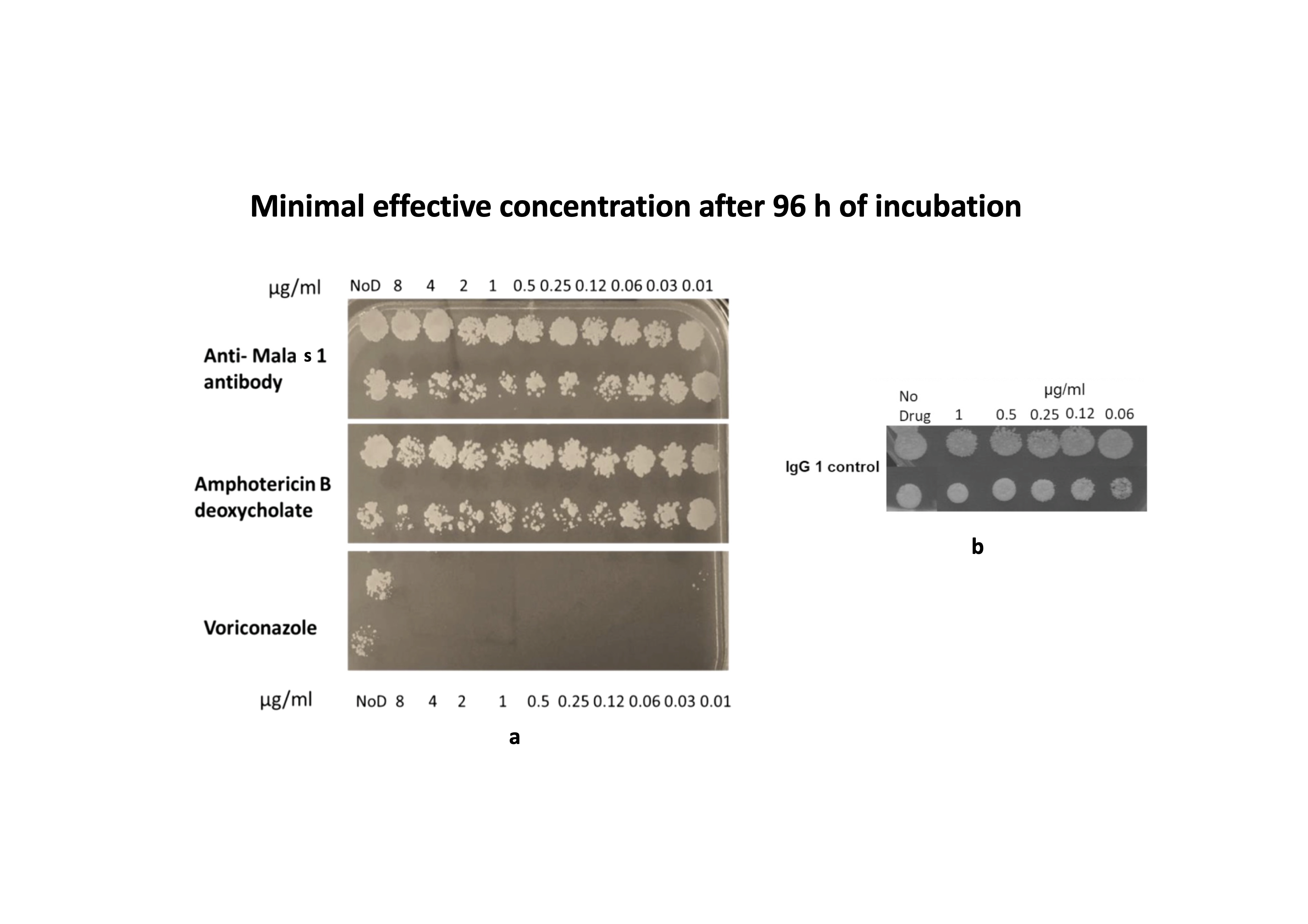

Supplement: foad028_Supplemental_Files [file foad028_supplemental_files.zip › Sup Fig 2.png]

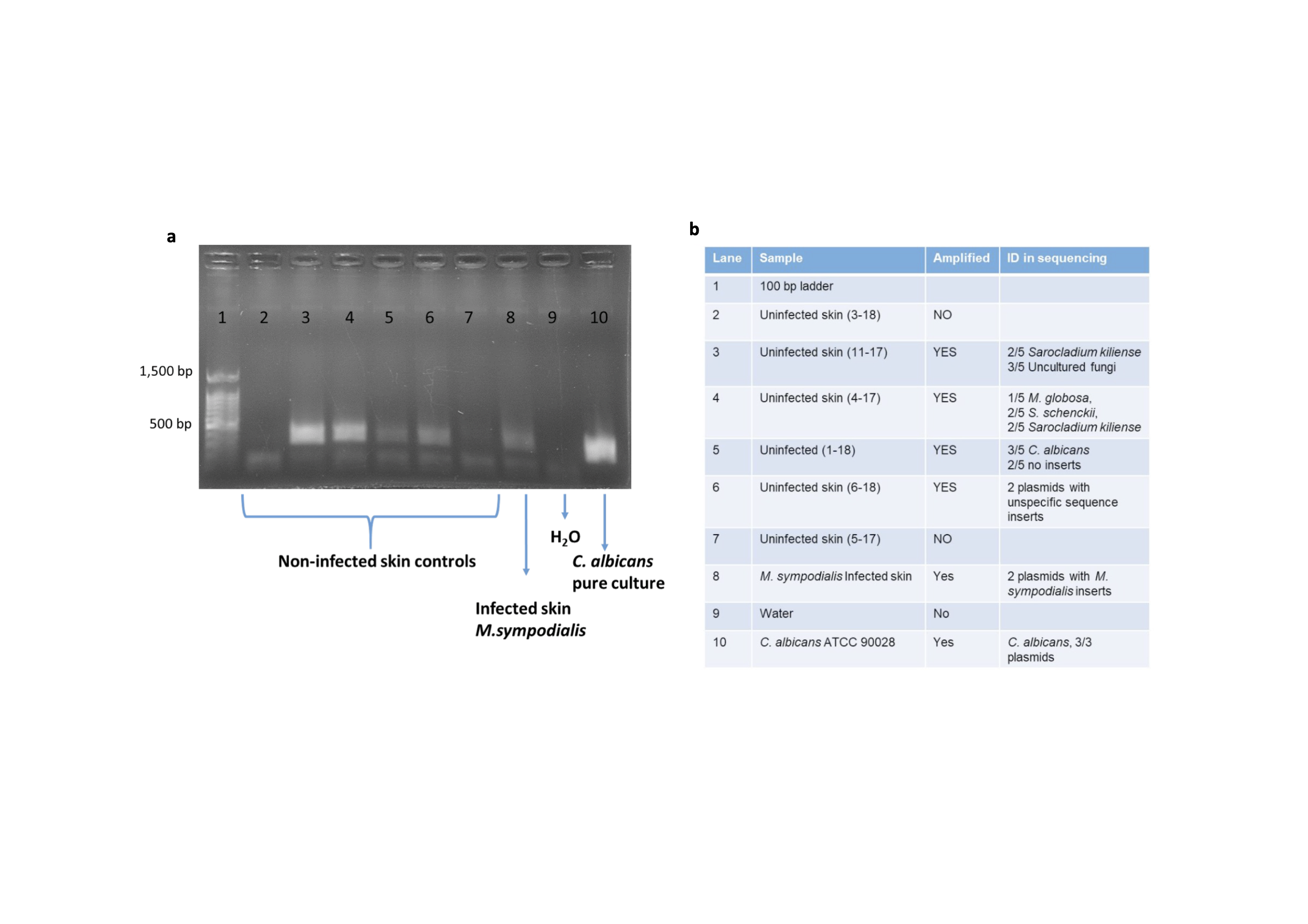

Supplement: foad028_Supplemental_Files [file foad028_supplemental_files.zip › Sup Fig 4.png]
